# Riverine Microplastic Quantification: A Novel Approach Integrating Satellite Images, Neural Network, and Suspended Sediment Data as a Proxy

**DOI:** 10.3390/s23239505

**Published:** 2023-11-29

**Authors:** Ahmed Mohsen, Ferenc Kovács, Tímea Kiss

**Affiliations:** 1 Department of Geoinformatics, Physical and Environmental Geography, University of Szeged, 6722 Szeged, Hungary; ahmed_mohsen250@f-eng.tanta.edu.eg (A.M.); kovacsf@geo.u-szeged.hu (F.K.); 2 Department of Irrigation and Hydraulics Engineering, Tanta University, Tanta 31527, Egypt

**Keywords:** Tisza River, machine learning, remote sensing, hydrological regimes, spatiotemporal distribution

## Abstract

Rivers transport terrestrial microplastics (MP) to the marine system, demanding cost-effective and frequent monitoring, which is attainable through remote sensing. This study aims to develop and test microplastic concentration (MPC) models directly by satellite images and indirectly through suspended sediment concentration (SSC) as a proxy employing a neural network algorithm. These models relied upon high spatial (26 sites) and temporal (198 samples) SSC and MPC data in the Tisza River, along with optical and active sensor reflectance/backscattering. A feedforward MLP neural network was used to calibrate and validate the direct models employing k-fold cross-validation (five data folds) and the Optuna library for hyperparameter optimization. The spatiotemporal generalization capability of the developed models was assessed under various hydrological scenarios. The findings revealed that hydrology fundamentally influences the SSC and MPC. The indirect estimation method of MPC using SSC as a proxy demonstrated higher accuracy (R^2^ = 0.17–0.88) than the direct method (R^2^ = 0–0.2), due to the limitations of satellite sensors to directly estimate the very low MPCs in rivers. However, the estimation accuracy of the indirect method varied with lower accuracy (R^2^ = 0.17, RMSE = 12.9 item/m^3^ and MAE = 9.4 item/m^3^) during low stages and very high (R^2^ = 0.88, RMSE = 7.8 item/m^3^ and MAE = 10.8 item/m^3^) during floods. The worst estimates were achieved based on Sentinel-1. Although the accuracy of the MPC models is moderate, it still has practical applicability, especially during floods and employing proxy models. This study is one of the very initial attempts towards MPC quantification, thus more studies incorporating denser spatiotemporal data, additional water quality parameters, and surface roughness data are warranted to improve the estimation accuracy.

## 1. Introduction

Plastic pollution in aquatic ecosystems is an emerging environmental issue worldwide [1]. Rivers transport plastics (1.2–2.41 million tons/year) accounting for 80% of ocean plastics, and it is expected to reach 2.75 million tons by 2040 [2]. Although several studies have investigated the spatiotemporal distribution, potential sources, pathways, and accumulation hotspots of riverine plastic, particularly microplastics (MPs: particle size < 5 mm), many aspects of MP transport are still unknown [3,4]. It is partly attributed to the fact that most MP studies have primarily relied on in situ measurements, which were limited in space and time. Hence, their sampling schemes were often insufficient for tracking the highly dynamic MP transport [1]. On the other hand, other studies developed numerical models, e.g., CaMPSim-3D [5] and INCA [6] to address the limitations of in situ measurements through providing highly-detailed spatiotemporal data. However, to build these models, many hydrological, morphological, meteorological, and MP characteristics data are needed, which may not be readily available for many rivers.

In the meantime, remote sensing could offer a more reliable and cost-effective monitoring solution owing to its large-scale coverage, frequent imaging, and extended time series. Additionally, it can provide valuable data for physically inaccessible river sections and during extreme weather conditions [7]. The revolutionary development in satellite constellation characteristics (i.e., high spatial, temporal, and spectral resolutions) pave the road toward the automatic detection of MPC in natural waters. However, this approach is still in its infancy, with conflicting findings regarding its applicability (Table 1). Bentley [8] asserted that MPs have a distinct spectral signature in the near-infrared (NIR) spectrum, with significantly higher reflectance than water. Thus, he developed a reflectance model based on the absorption and backscattering coefficients of plastics and water at the 748 nm and 869 nm wavelengths, along with in situ MPC measurements at the Great Pacific Garbage Patch. Meanwhile, Hu [9] contended that remote sensing of MPC in aquatic ecosystems is not feasible using any current or futuristic passive sensors. He argued that the MPC influence on the sensor signal is 60 times less than the desired signal (i.e., subpixel coverage of 0.2%), even if the MPs aggregated at the maximum reported concentration in the literature, and lower than the sensor noise by 20 times (signal-to-noise ratio SNR of 200). Accordingly, our study aims to resolve this discrepancy by analyzing the relationship between MPC and the reflectance/backscattering of the various passive and active space-borne sensors.

Remote sensing studies that have successfully estimated MPC in aquatic ecosystems mostly relied on simulated proxy measurements. For instance, Davaasuren et al. [10] hypothesized that surfactants (i.e., carbon molecules with short or complex chains) which are released by microbial colonization of MP particles, could be employed as a proxy for MP abundance due to their capability to affect the fluid dynamic characteristics, e.g., viscosity and surface tension. Their assertion was confirmed by noticing dark signatures in SAR images covering ocean gyres in the North Pacific and North Atlantic Oceans, which are presumably linked to microbiological activity caused by MP colonization. Similarly, Evans and Ruf [11] have formulated an empirical algorithm for estimating MPC by SAR images in oceans based on their observations that the presence of surfactant lowers wind-driven water surface roughness and that the degree of suppression is linked to MPC. However, establishing such a relationship in rivers is challenging, due to differences in magnitudes and patterns of wind-driven waves and ripples in rivers than the ocean, caused by faster currents and higher turbulence in rivers, besides its limited width and riparian vegetation along the floodplain [12]. Alternatively, we hypothesized that SAR images could directly estimate MPC. Particularly, because He et al. [13] stated that the water dielectric constant is associated with the particulate material concentration and SAR images are highly sensitive to dielectric variations [14]. Therefore, our study aims to evaluate their direct correlation.

Light-responsive water components, e.g., SSC and chlorophyll-a, could be employed as effective proxies for MP estimation in rivers, given their good correlation with MPC and the ability of optical sensors to estimate them accurately [15,16]. Mercedes et al. [17] reported that the spatial distribution of chlorophyll-a is related to MPC, as usually, the algae cling to the floating plastic particles. Piehl et al. [16] revealed a good association among MPC and colored dissolved organic matter (R^2^ = 0.60), chlorophyll-a (R^2^ = 0.57), and SSC (R^2^ = 0.38) in the Trave River (Germany). Similarly, a good relationship with a higher magnitude (R^2^ = 0.65) was found between SSC and MPC in the Langat River (Malaysia) [15]. Atwood et al. [2] disclosed a good agreement between the MP accumulation map generated by the remote sensing-based SSC model and the in situ measurements of MPC samples collected from the sediment of the Po River (Italy).

**Table 1:** A review of literature on utilizing satellite data for quantifying microplastic concentration (MPC) in aquatic environments worldwide.

Reference	Location	Satellite Sensor	Developed Model/ Type of Analysis	Application of a Proxy	Applicability of Satellite-Based MPC Estimation
Bentley [8]	Great Pacific Garbage Patch	MODIS Aqua	Reflectance model using 748 nm and 869 nm wavelengths	No	Yes
Hu [9]	Several marine environments	Sentinel-2	Sensitivity and spectral analysis	No	Impossible
Davaasuren et al. [10]	North Pacific and North Atlantic Oceans	Sentinel-1A and COSMO-SkyMed	Detecting the influence of surfactant (related to MPC) on wind-driven surface roughness	Surfactants and bio-film	Probably by SAR images
Evans and Ruf [11]		CYGNSS bistatic radars	Empirical model based on surfactant (related to MPC) influence on wind-driven surface roughness		Yes
Piehl et al. [16]	Trave, Elbe, and Po Rivers	Landsat 8	Empirical models between MPC and active water constituents	Colored dissolved organic matter, chlorophyll-a, and SSC	Probably
Atwood et al. [2]	Po River		Remote sensing-based SSC model as a proxy for MPC	SSC	Yes (with some limitations)

This study focuses mainly on SSC as a proxy, since it is a commonly measured parameter in rivers worldwide and shares several characteristics with MP particles. The occurrence of MPs in rivers is related to fine-grained sediments [18]; thus, the distribution of both particles is governed by similar mechanisms. Additionally, they are transported with the same modes and particles exhibit vertical stratification determined by the grain density and size [18]. However, there are differences between MP and natural SS in terms of their densities, sources, shapes, persistence period, fragmentation, and degradation rates that complicate their relationship [18]. Therefore, we hypothesized that their relationship might be altered temporally due to seasonal variations in hydrological, hydraulic, and vegetation conditions and spatially due to variations in their spatial sources (e.g., tributaries, wastewater treatment plants (WWTP), industrial effluents, and mining activities). Unfortunately, most of the former studies assessed their relationship at limited spatial and/or temporal scales (e.g., snapshot measurements [16] and monthly measurements [15]), thus the established relationships may have some degree of uncertainty. Hence, our study aims to overcome this issue through intensive in situ SSC and MPC measurements and individual assessment for every hydrological condition separately.

Satellite sensors have the potential to offer valued data on origins, pathways and destinations of riverine MP. However, to our knowledge, no study has yet attempted to investigate their ability as a monitoring tool in rivers, as the studies focused on the marine environment [10,11] (Table 1). Therefore, the main aim of the present study is to address this research gap by developing a neural network-based model to estimate MPC in rivers based on varying optical [Sentinel-2 (S-2) and PlanetScope (PS)] and active [Sentinel-1 (S-1)] satellite sensors. The goals of the study are: (1) to evaluate the direct correlation between SSC/MPC and reflectance/backscattering of the designated sensors, (2) to test the efficiency of SSC (measured and estimated by satellites) as a proxy for estimating MPC considering both overall and individual hydrological conditions, (3) to develop neural network-based models to estimate SSC/MPC by various sensors and compare their estimation accuracy, and finally (4) to identify the most important bands for SSC/MPC estimation for each sensor.

The article is structured into four key sections. Section 2, Material and Methods, describes the study area (Tisza River), the collected in situ and remote sensing data, along with their analysis, and the developed direct and indirect models. Section 3 and Section 4 present Results and Discussions on the developed models, revealing their spatiotemporal potentials and implications. Section 5 concludes the entire study.

## 2. Materials and Methods

### 2.1. Study Area

**Figure 1:**
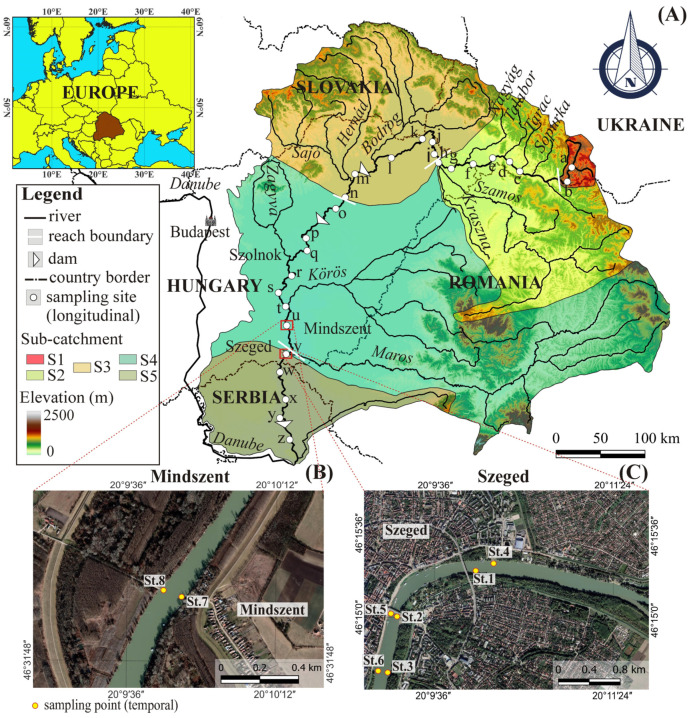
The catchment of the Tisza River is in Central Europe. The sampling sites (a–z) cover the whole length of the Tisza (962 km) (**A**). Two sampling sites for detailed temporal measurements were selected at Mindszent (**B**) and Szeged (**C**).

The Tisza River (Central Europe) was selected for the case study (Figure 1). The catchment area (157,000 km^2^) of this lowland river belongs to the catchment of the Danube, contributing to 13% (58–4346 m^3^/s) of the Danube’s discharge [7]. Most of the discharge (93.8%) springs from the hilly sub-catchments in Romania (56.4%), Ukraine (26.6%), and Slovakia (10.8%), while only 6.2% originates from the plains of Hungary and Serbia [19,20]. Two floods usually characterize the regime of the Tisza, one from March to April, and another from June to July, throughout the rest of the year the Tisza has low stages [21].

Our study covered the entire river from Ukraine to Serbia (962 km); yet, two towns (i.e., Mindszent and Szeged) were selected on the lowland section for temporal measurements (Figure 1). The Tisza was divided into three reaches and five sections (S1–S5) based on their longitudinal hydrological, morphological and meteorological characteristics.

The Upper Tisza (962–688 km) is located in the NE Carpathian Mountains (Figure 1A). Its upper section (S1) is located in an incised valley with steep slopes, and the river has a high flow velocity (Appendix A: Table A1). Towards downstream (S2 section) the channel gradually widens, the slope declines (by 97%), and the flow velocity drops (by 60%); however, the discharge increases by nine times. The Upper Tisza transports a large amount of bed load and a relatively low amount of suspended material (Appendix A: Table A1). In the valleys of the Carpathian sub-catchments, the significant amount of mismanaged communal waste (Ukraine: 12.3 million tons/year; Romina: 5.4 million tons/year) is coupled with a very limited recycling ratio (Ukraine: 1%; Romania: 12%) [22]. Additionally, most of the wastewater is left untreated (Ukraine; 69%; Romania 48%) [23].

The Middle Tisza (688–177 km) comprises two sections. The S3 drains mainly the hilly Slovakian and Hungarian sub-catchments, while the S4 drains the hilly Romanian and the lowland Hungarian sub-catchments (Figure 1A). Along these sections the slope decreases significantly (S3: 77%; S4: 33%) as well as the flow velocity (S3: 70%; S4: 50%) compared to the previous section (Appendix A: Table A1). The bed load transport also decreases between S2 and S3 (61%); however, it slightly increases (25%) towards S4. Meanwhile, the mean water discharge increases (S3:1.7-folds; S4: 1.5-folds) and the suspended sediment load becomes dominant (S3: 5.6-fold increase; S4: 2.4-fold increase). Within this reach, the sediment and MP transport are heavily affected by tributaries and dams. Our frequent temporal measurements of SSC and MPC were performed in this reach at the Mindszent site (‘u’; 217 km) (Figure 1A,B). Here, the channel is 108 m wide and 11 m deep, and the maximum water stage fluctuation is 13.5 m [24]. In the Middle Tisza sub-catchment, the low annual communal waste production (3.5 million tons) is accompanied with a high recycling ratio (36%), and 83% of residences are linked to wastewater systems [22].

The Lower Tisza (177–0 km) is mainly located in Serbia (Figure 1A). Since its hydro-morphological parameters are quite uniform, it was considered as one section (S5). This impounded reach has the lowest slope and flow velocity, and consequently, the bed load transport is also limited (Appendix A: Table A1). Meanwhile, it has the highest suspended sediment load and water discharge. The Szeged site (‘v’), where additional temporal SSC measurements were performed, is located upstream of the reach (Figure 1A,C). The sampling area extended from the Tisza-Maros confluence to 4.2 km long (177–172.8 km). Here, the channel is wide (164.1 m) and deep (14 m) [24]. Along the Lower Tisza, the waste management practices are quite poor, since only 1% of the communal waste production (3 million tons/year) is recycled [22]. Furthermore, a low proportion (56%) of households is connected to waste water treatment plants (WWPS), and most of the collected water (71%) is untreated [23].

### 2.2. In Situ Measurements

The spatial and temporal distribution of SSC and MPC in the Tisza River was investigated by intensive in situ measurements. The spatial samplings were conducted at 26 sites, from Ukraine to Serbia (Figure 1A). These measurements were made at low stages (August 2021 and July 2022); however, the sites in Ukraine (‘a–e’) were excluded in 2022, because of the war. The MPC was measured in 2021 and 2022, while the SSC was measured only in 2022. The coordinates of the sampling sites were determined by GPS in the field.

**Figure A1:**
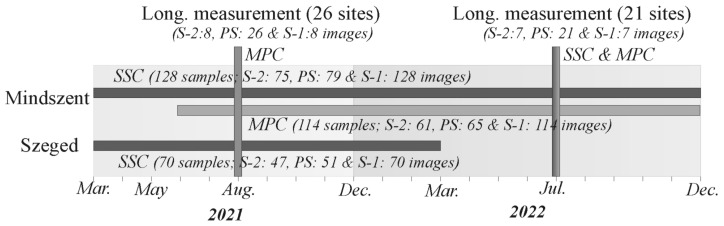
Spatiotemporal monitoring scheme for suspended sediment (SS) and microplastic (MP) concentrations in the Tisza River.

At Mindszent (site ‘u’; Figure 1B), the SSC and MPC were observed every five days: the SSC was measured for almost two years (March 2021–December 2022; 128 samples), but the MPC measurements started later due to technical difficulties (May 2021–December 2022; 114 samples) (Appendix A: Figure A1). Additional SSC measurements were made in Szeged (site ‘v’; Figure 1C) every five days for one year (March 2021–March 2022; 70 samples) (Appendix A: Figure A1).

The SSC was monitored by collecting 1.5 L of water from the surface of the river. The MPC was monitored by pumping 1 m^3^ of water (20–30 cm depth) passing through metal sieves (90–2000 µm); then, the residuals were rinsed to glass jars (350 mL). The longitudinal measurements for SSC and MPC were performed from one side of the riverbanks. Meanwhile, the temporal measurements in Mindszent for SSC were accomplished from near-bank points (St.7 and St.8; Figure 1B), and for MPC across the whole width of the river from a moving ferry. Similarly, the SSC measurements in Szeged were performed from the near-bank at six points (St.1–St.6) (Figure 1C).

The hydrological condition of the river was determined through water stage (H) data measured daily at Mindszent (site ‘u’) by the local Hydrological Water Directorate (ATIVIZIG).

### 2.3. Laboratory Analysis

Our study adopted the evaporation method for estimating SSC since the natural sediment of the Tisza is dominated by fine grains [25], and occasionally the concentration exceeds the applicability limit (200 mg/L) of the infiltration method for fine grain samples [26]. The measurement process followed the ASTM D3977-97 (A) standards [27]. Thus, samples were left for at least one week to allow sediment to settle. Subsequently, the excess water was removed and sediment were dried at 105 °C for 24 h. The dried sediment was weighed, and the SSC (g/m^3^) was estimated by dividing the dry weight of the sediment by the volume of the collected water sample (1.5 L).

The collected samples for measuring MPC were divided into low and high SS content samples. The elevated SSC in samples with high content was eliminated by applying a density separation process using zinc chloride (1.8 g/cm^3^) [21]. Then these samples were digested by hydrogen peroxide (30%) for 48 h [4] to remove the organic content. Meanwhile, samples with low SSC were just digested following Balla et al. [4]. Finally, all samples were rinsed into a petri dish and dried for 36 h at 60 °C, to be prepared for the subsequent identification and counting process.

The MP counting was accomplished by visual identification using a digital microscope (Ash Inspex II, Dublin, Ireland) under a magnification rate of 60×. The visual identification adhered to the framework outlined by Balla et al. [4] and Kiss et al. [21]. For instance, a particle was considered as MP if (1) no visible cellular structures were found; (2) the fiber maintained a consistent color and thickness throughout; (3) it responded to the hot needle test, retaining its rigid shape upon movement.

To validate the identification accuracy, the ATR-FTIR (Shimadzu Infinity 1s device, Santa Clara, CA, USA) was employed in the range 400–4000 cm^−1^ utilizing the database of Shimadzu Standard. A total of 150 randomly chosen items were analyzed, resulting in an identification precision of 98%. The MP particles were categorized into fragment, fiber, and bead [4], and the MPC was expressed in item/m^3^.

During the MP extraction process, airborne fiber contamination is common [28]; therefore, the contamination mitigation measures of Balla et al. [4] were applied during the laboratory work. As complete contamination prevention is challenging, a fifth blank sample was included for every four samples, and it underwent identical steps as the original samples. Fibers dominated the blank samples with a mean of 5.7 ± 3.4 item/sample, which forms 8.6% of the original samples. Therefore, the MP content in each original sample was adjusted by deducting the identified MPs in the blank sample from its total count.

### 2.4. Remote Sensing Data

#### 2.4.1. Passive Sensors

The multi-spectral Sentinel-2 MSI and high spatial resolution PlanetScope satellites were employed. Altogether, 122 Sentinel-2A-B images acquired from the Copernicus Open Access Hub (https://scihub.copernicus.eu/, accessed on 1 October 2023) were utilized. These images covered the entire river in August 2021 (8 images) and July 2022 (7 images) and the sites at Mindszent (March 2021–December 2022; 75 images) and Szeged (March 2021–March 2022; 47 images). The images were cloud-free at the corresponding sites and synchronous with the in situ sampling dates. Sentinel-2 provides images with 13 spectral bands (wavelength: 443–2190 nm) with 10–60 m spatial resolution (Appendix A: Table A2), and 3–5-day temporal resolution.

PlanetScope satellite constellation, operated by Planet Labs, has more than 180 sun-synchronous CubeSats, which offer daily images to almost all the globe [29]. Altogether, 177 PlanetScope images (https://www.planet.com/explorer, accessed on 1 October 2023) were employed, of which 26 images covered the longitudinal measurements along the Tisza in August 2021, 21 images in July 2022, and further 79 images covered the temporal samplings in Mindszent, and 51 images in Szeged. These images were captured by the Super Dove sensor (PSB.SD) (Tomball, TX, USA) which provides eight bands (wavelength: 431–885 nm), in a 3 m spatial resolution [29] (Appendix A: Table A2). The images were synchronous with the in situ sampling dates with a maximum shifting of ±1-day timeframe.

#### 2.4.2. Active Sensor

Altogether, 175 dual-polarimetric Sentinel-1A-B SAR images (C-band; 5.405 GHz) were obtained through the Copernicus Open Access Hub. The images covered the longitudinal sites in August 2021 (eight images) and July 2022 (seven images), and the temporal measurements at Mindszent (March 2021–December 2022; 128 images) and Szeged (March 2021–March 2022; 70 images). The Ground Range Detected (GRD) Level 1 data with the Interferometric Wide Swath (IW) mode were employed since it is commonly used in water bodies’ backscattering investigations [14]. The spatial resolution of the images is 10 m, acquired every 5 days in two channels [cross- (VH) and co- (VV) polarization].

### 2.5. Pre-Processing of Remote Sensing Data

#### 2.5.1. Passive Sensors

Both the Sentinel-2 (Level 2A) and PlanetScope (Level 3B) images were already radiometrically and atmospherically corrected. The bands of Sentinel-2 were consistently resampled to 10 m spatial resolution. The Normalized Difference Water Index (NDWI) [30] and OTSU automatic thresholding algorithm [31] were employed to provide a water mask based on Sentinel-2 images. The OTSU algorithm aimed to find the optimal NDWI threshold that maximizes the variance between water class and background, based on the image histogram. The derived mask was applied to extract the river water not only in Sentinel-2 but also in PlanetScope images. A region of interest of 2 × 2 pixels (i.e., 20 × 20 m) in Sentinel-2 and 6 × 6 pixels (i.e., 18 × 18 m) in PlanetScope were identified around every sampling point and the bands’ reflectance of both satellites was extracted. The Sentinel Application Platform (SNAP) software (version 8.0) and Python 3.0 were utilized to analyze the images.

#### 2.5.2. Active Sensor

The Sentinel-1 images were clipped to the extent of the corresponding measuring site to minimize the pre-processing time. Then, they were radiometrically calibrated converting the backscattering intensity to normalized radar cross section (sigma 0: σ_0_) based on the incidence angle and sensor-specific characteristics. The speckle filter (Lee sigma; window of 7 × 7 pixels; sigma value of 0.9) was employed to remove speckle noise. Finally, the images were geocoded to WGS 84/UTM zone 34N and corrected for geometric distortions using the SRTM 1sec HGT digital elevation model and the bilinear interpolation resampling method. To improve image visibility, the σ_0_ was converted to a log scale expressed as dB. In the same vein, the river water was extracted using the Sentinel-2 water mask, and a region of interest of 2 × 2 pixels (i.e., 20 × 20 m) was identified around every sampling site, where the backscattering of VV and VH channels was extracted.

### 2.6. Correlation Analysis

The strength of correlation among (1) measured SSC and MPC and (2) reflectance of Sentinel-2 and PlanetScope bands was investigated. In addition, the strength of the correlation between SSC and MPC themselves was also evaluated, considering all hydrological periods collectively and individually (i.e., low stages and rising, peak, and falling phases of floods). Since the daily water change level during low stages was ≤15 cm/day, it was considered as a threshold between low stages and floods. Furthermore, floods were divided into rising (positive water stage difference), peak (subtle water stage difference at the summit of the hydrograph), and falling (negative water stage difference) phases. The assumption of normality for the SSC and MPC data was not met (*p* < 0.001); thus, the correlation analysis was performed by the Spearman’s rank-order correlation test. The normality test and correlation analysis were conducted with the SPSS (The International Business Machines Corporation (IBM); Armonk, NY, USA); statistical software V26.0).

The reliability of SAR images to detect changes in the dielectric constant of water influenced by SSC/MPC was evaluated, especially because it was reported that dielectric constant is related to the suspended particulate material content [32]. Hence, the correlation between SSC and MPC and the backscattering of SAR channels were investigated by Spearman’s rank-order correlation test as a primary step toward SSC/MPC estimation by SAR images in rivers.

### 2.7. Remote Sensing of Suspended Sediment (SS) and Microplastic (MP) Concentrations

Regression models for SSC and MPC were developed considering the reflectance of Sentinel-2 and PlanetScope images as well as the backscattering of Sentinel-1 images. The models were developed based on the MLP neural network (regression module) since it is very efficient to deal with non-linear and high-dimensional problems. For Sentinel-2 images, all but bands with a low spatial resolution of 60 m (i.e., B1, B9, and B10) and with low sensitivity to SSC (i.e., B11 and B12) were employed as independent variables. Similarly, all but B1 and B8 were employed for the regression analysis in PlanetScope. Meanwhile, both channels of Sentinel-1 images (i.e., VV and VH) were used.

The k-fold cross-validation was applied to divide the data (i.e., bands and SSC/MPC) to five training and validating folds. This approach is quite useful to mitigate the chances of the model’s overfitting and random sampling biases, as it ensures that the model was trained and validated at different datasets. The generalization potential of the models was assessed through some spatiotemporal data that were excluded from the training/validation dataset and tested against the models’ predictions. The temporal testing data covered all hydrological scenarios (i.e., low stages (7 August 2021 and 3 June 2022), rising (4 April 2022 and 6 October 2022), peak (25 December 2022 and 4 May 2022) and falling (9 May 2022 and 16 October 2022) phases of floods), while the spatial data covered eight sites “i–p” upstream of the Kisköre Dam measured in July 2022.

**Figure A2:**
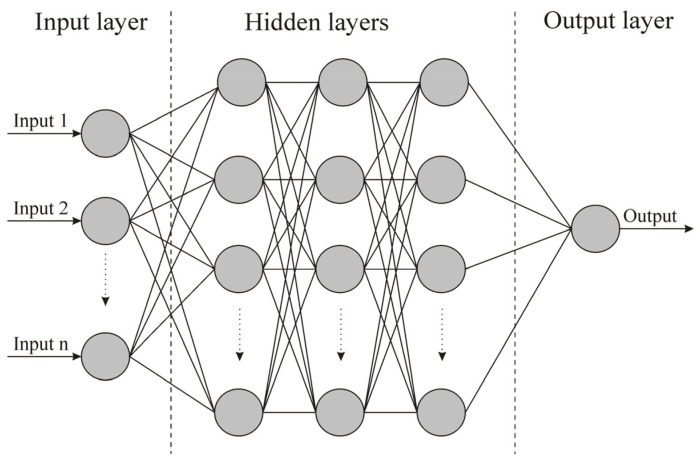
The architecture of the MLP neural network used to build the suspended sediment (SS) and microplastic (MP) concentration models based on Sentinel-2, PlanetScope, and Sentinel-1 sensors.

The multilayer perceptron (MLP) feedforward neural network was selected to develop the SSC/MPC models leveraging its potential to learn complex patterns from training data. The main structure of the models was a network consisting of interconnected neurons organized in input, hidden, and output layers (Appendix A: Figure A2). Every neuron performs a computation and passes its result to the next layer. The neurons are linked through weights that are randomly initiated and updated during the training process to minimize the loss function (RMSE; Equation (1)) based on the selected solver (e.g., ‘adam’, ‘sgd’, and ‘lbfgs’). The activation function (e.g., ‘identity’, ‘logistic’, ‘tanh’, and ‘relu’) introduces non-linearity into the network and give it a chance to learn complex patterns [33,34,35].

Since the model’s hyperparameters control its prediction accuracy significantly, the Optuna open-source Python library [36] was used for their optimization based on the concepts of the Bayesian optimization technique. Specifically, we employed the tree-structured parzen estimator (TPE) algorithm which models the objective function as a probability distribution. This algorithm uses a tree structure to explore the search space of hyperparameters and update the distribution based on the observed results. Our study considered a set of hyperparameters for the SSC/MPC models, including hidden layer sizes, activation function, solver, alpha, learning rate, maximum iteration, and batch size, and defined their search spaces. The RMSE was considered as the model’s score, and the optimizer was adjusted to minimize the score over 300 trials. The best-performing combination of hyperparameters (Table 2) was used to train the final SSC/MPC models. Two additional evaluation metrics: coefficient of determination (R^2^) and mean absolute error (MAE) (Equations (2) and (3)) were employed to assess the performance of the models.

The contribution of bands in the performance of the derived models was determined by the Shapley additive explanations (SHAP) Python library [37]. This technique is based on the cooperative game theory concept, as the importance of each band is expressed through a SHAP value. This value is estimated by calculating the contribution of a particular band in the model’s prediction accuracy with and without its existence across all permutations. The kernel optimization algorithm was applied, and the findings were visualized by a violin plot that showcases the features sorted in descending order of importance.

(1) $$RMSE=\sqrt{\frac{\sum_{i=1}^{n}{\left(y_{i}-{\hat{y}}_{i}\right)}^{2}}{n}}$$

(2) $$R^{2}=1-\frac{\sum_{i=1}^{n}{\left(y_{i}-{\hat{y}}_{i}\right)}^{2}}{\sum_{i=1}^{n}{\left(y_{i}-\bar{y}\right)}^{2}}$$

(3) $$MAE=\frac{1}{n}\sum_{i=1}^{n}\left|y_{i}-{\hat{y}}_{i}\right|$$

where: $y_{i}$: observed value, ${\hat{y}}_{i}$: predicted value by a model, $\bar{y}$: mean observed, and n: number of observations.

### 2.8. Suspended Sediment Concentration as a Proxy for Microplastics

Based on the results, the MPC models developed by passive and active sensors showed comparatively low prediction accuracy. In the meantime, SSC exhibited a good correlation with MPC, especially during floods. This correlation arises due to the similarity of their sources, especially during floods, when the resuspension of deposited materials and runoff events raise their concentrations simultaneously. However, during low stages, these sources terminate, and non-flood sources (e.g., bank erosion, WWTPs, and tributaries) take precedence. Thus, we supposed that SSC can be employed as a viable proxy for MPC. Hence, regression models were built between both variables considering all hydrological conditions and individually (i.e., low stages and rising, peak, and falling phases of floods). To simplify the regression complexity, simple regression techniques, e.g., linear and polynomial regressions were tested. These proxy models were developed in the SPSS software.

## 3. Results

### 3.1. Spatiotemporal Distribution of Suspended Sediment (SS) and Microplastic (MP) Concentrations in the Tisza River

**Figure 2:**
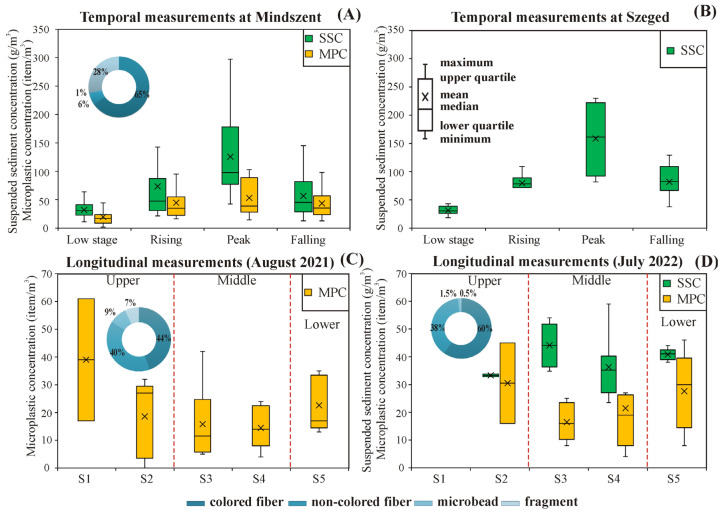
Temporal changes in suspended sediment concentration (SSC) and microplastic concentration (MPC) measured at Mindszent (**A**) and Szeged (**B**); and longitudinal changes along the river sections (S1–S5) in August 2021 (**C**) and in July 2022 (**D**).

The SSC and MPC revealed similar temporal changes as the hydrograph, with low concentrations during low stages (mean SSC: 32.2 ± 12.1 g/m^3^; mean MP: 20 ± 14.2 item/m^3^) and elevated concentrations during floods (mean SSC: 71.9 ± 59.4 g/m^3^; mean MP: 45.4 ± 28.2 item/m^3^) (Figure 2A). Considering the phases of flood waves, the mean SSC varied greatly (i.e., rising: 73.4 g/m^3^; peak: 125.7 g/m^3^; falling: 56.7 g/m^3^); however, this variability was less pronounced in MPC (i.e., rising: 44.7 item/m^3^; peak: 53.3 item/m^3^; falling: 43.5 item/m^3^). A comparable distribution pattern of SSC was noticed in Szeged as in Mindszent; however, the mean concentration in Szeged was 1.3 times higher than in Mindszent (Szeged: 66.8 g/m^3^; Mindszent: 52 g/m^3^) (Figure 2A,B). The MP morpho-types had a similar frequency in all samples, e.g., fibers (81%) dominated the MP composition with a relatively high abundance of fragments (28%) and a subtle abundance of microbeads (1%) at the Mindszent site.

The SSC and MPC varied along the Tisza in both years. Higher variability was observed in MPC than in SSC (i.e., SSC range: 33.3–44.12 g/m^3^; MPC range: 14.5–39 item/m^3^); however, they had no clear downstream trend (Figure 2C,D). The mean MPC along the river in 2022 (22.4 ± 14.8 items/m^3^) was 18% higher than in 2021 (19 ± 13.4 item/m^3^), which was also noticed during the temporal monitoring in Mindszent (2021: 25 ± 21.1 item/m^3^; 2022: 36 ± 30.5 item/m^3^). The most polluted sections were the upper (S1 and S2) and lower (S5) sections. In the meantime, the SSC distribution showed an adverse pattern, as the highest concentrations were measured in the Middle Tisza (S3: 44.12 g/m^3^; S2: 33.3 g/m^3^) (Figure 2D). The fibers dominated the longitudinal measurements (2021: 84.6%; 2022: 98%) with a negligible presence of fragments and microbeads.

### 3.2. Correlation and Spectral Signature

**Figure 3:**
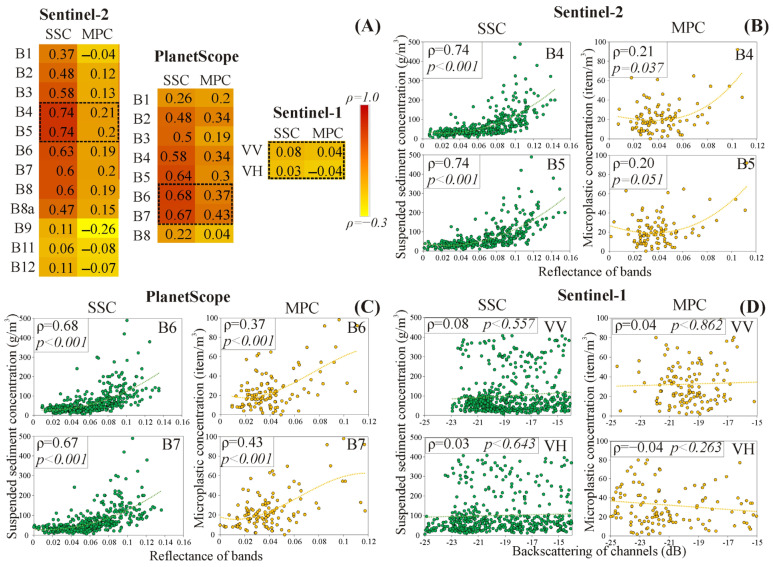
The Spearman’s rank-order correlation coefficient among suspended sediment concentration (SSC)/microplastic concentration (MPC) and reflectance/backscattering of Sentinel-2, PlanetScope, and Sentinel-1 sensors (**A**). Scatter plots for the two bands with the highest correlation coefficients were represented (**B**,**C**), as well as the VV and VH channels of Sentinel-1 (**D**).

The bands of Sentinel-2 and PlanetScope showed negligible (ρ = 0.04) to very strong (ρ = 0.74) correlations with the SSC and MPC, while the backscattering of Sentinel-1 channels exhibited only negligible correlations (ρ = 0.03–0.08) (Figure 3A). Remarkably, the SSC showed a higher correlation with the Sentinel-2 and PlanetScope bands than the MPC. The highest correlation of SSC was found with the Sentinel-2, while for the MPC it was found with the PlanetScope. The spectral regions encompassing visible and near-infrared (i.e., Sentinel-2: B2-B8a; PlanetScope: B2-B7) were sensitive to SSC and MPC changes, particularly bands B4 and B5 in Sentinel-2 and their counterparts B6 and B7 in PlanetScope. Although MPC showed its highest correlation with B9 (in Sentinel-2; ρ = −0.26), this band is not suitable for MP detection due to its low spatial resolution (60 m) and high sensitivity to water vapor content, which interprets its negative correlation. The scatter plots of the highly correlated bands with the SSC and MPC (i.e., B4 and B5 in Sentinel-2 and B6 and B7 in PlanetScope) suggest a non-linear relationship (Figure 3B,C), while no correlation was noticed with the Sentinel-1 channels (Figure 3D).

Considering band compatibility between Sentinel-2 and PlanetScope (Appendix A: Table A2), most bands showed similar correlation patterns with SSC and MPC. For instance, B8a (Sentinel-2) showed high correlations with SSC and MPC, while its counterpart B8 (PlanetScope) demonstrated lower correlations. This can be interpreted due to spectral (bandwidth and signal-to-noise ratio SNR) and spatial resolution differences between both sensors. Furthermore, the shorter revisit time of PlanetScope provided more images than Sentinel-2, consequently, the Spearman’s correlations were calculated based on slightly different datasets.

**Figure 4:**
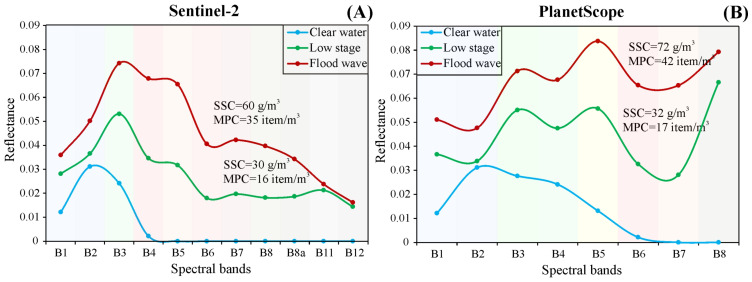
Spectral signature of river water during the low stage and flood wave in comparison to clear water based on the mean reflectance of the Sentinel-2 (**A**) and PlanetScope (**B**) bands.

The mean reflectance of the Sentinel-2 and PlanetScope bands of river water (during low stages and floods) were depicted in comparison to the spectral signature of clear water (Figure 4). The spectral signature of river water (both hydrological conditions) deviated from clear water in the red and NIR bands. Specifically, a negligible reflectance of clear water at these bands was noticed in contrast to an elevated reflectance of river water. Notably, the spectral pattern of river water during low stages and floods was very similar, differing primarily in their magnitudes. The greatest reflectance difference between clear and river water occurred at the B3, B5, and B6 bands in Sentinel-2 and the B5, B6, and B7 bands in PlanetScope, which interprets their elevated correlations with the SSC/MPC (Figure 3A). Furthermore, these bands have the widest reflectance difference between the low stages and flood waves, referring to their higher sensitivity to SSC/MPC changes.

**Figure 5:**
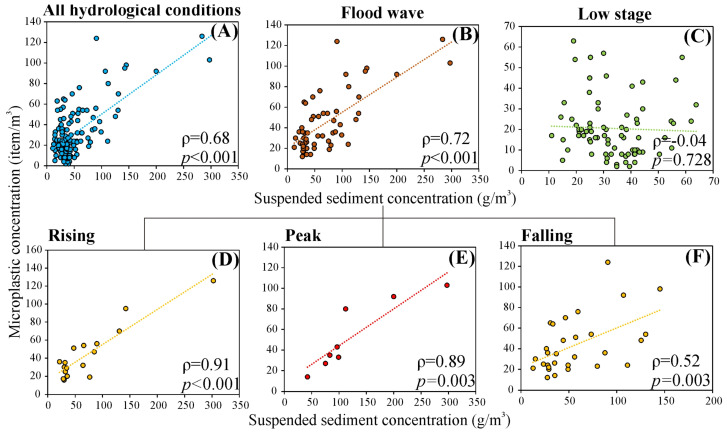
The Spearman’s rank-order correlation coefficient among suspended sediment concentration (SSC) and microplastic concentration (MPC) for different hydrological scenarios: all hydrological conditions (**A**), flood wave (**B**), low stage (**C**), and individual flood phases (i.e., rising, peak and falling) (**D**–**F**).

A strong positive correlation was revealed between SSC and MPC considering all spatiotemporal measurements (ρ = 0.68; Figure 5A). However, the observed correlation during flood waves (ρ = 0.72) markedly exceeded that of during low stages (ρ = −0.04; Figure 5B,C). It is noteworthy that during extremely low MPC, the correlation strength is likely to remain negligible. Considering flood waves, both variables were very strongly correlated during the rising (ρ = 0.91) and peak (ρ = 0.89) periods, while their correlation declined slightly to strong during the falling limb (ρ = 0.52; Figure 5D–F). These promising results demonstrate the potential of SSC as a viable proxy for MPs, especially during floods.

### 3.3. Suspended Sediment and Microplastic Concentration Models

#### 3.3.1. Remote Sensing-Based Models

**Figure 6:**
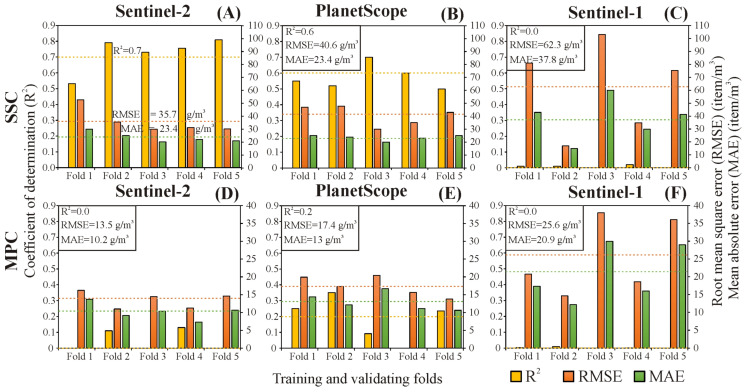
Evaluation metrics (i.e., R^2^, RMSE, and MAE) for the suspended sediment (SS) and microplastic (MP) concentration models based on Sentinel-2 (**A**,**D**), PlanetScope (**B**,**E**), and Sentinel-1 satellites (**C**,**F**) represented for the five data folds as well as their mean.

The evaluation metrics (i.e., R^2^, RMSE, and MAE) of the remote sensing-based SSC and MPC models were depicted for the five data folds as well as their mean (Figure 6). The passive sensors demonstrated the best metrics (Figure 6A,B,D,E), while the active sensor exhibited the worst metrics (Figure 6C,F). Among the passive sensors, the best-performing SSC model was achieved by Sentinel-2 (R^2^ = 0.7; RMSE = 35.7 g/m^3^ and MAE = 23.4 g/m^3^; Figure 6A); meanwhile, its counterpart in MPC was by PlanetScope (R^2^ = 0.2; RMSE = 17.4 g/m^3^ and MAE = 13 g/m^3^; Figure 6E); though, the estimation accuracy for MPC was still relatively low. The evaluation metrics for the passive-sensor-based models were consistent across the data folds, especially for SSC models (Figure 6A,B), indicating that the models were well-trained and performed consistently across the various datasets.

#### 3.3.2. Contribution of Bands in the Developed Models

**Figure 7:**
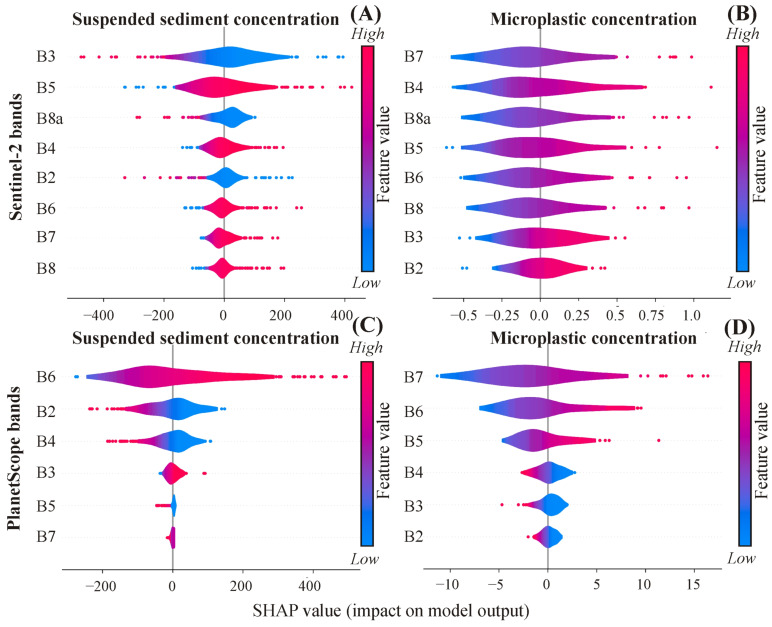
Violin plots reveal the contribution order of the bands of the Sentinel-2 (**A**,**B**) and PlanetScope (**C**,**D**) for suspended sediment concentration (SSC) and microplastic concentration (MPC) models expressed in a SHAP value.

Since the active-based models demonstrated notably low estimation accuracy (Figure 6C,F), the SHAP analysis was solely conducted on the passive-based models (Figure 7). The order of the most influential bands identified by the SHAP analysis did not precisely align with that indicated by the correlation matrices (Figure 3A and Figure 7). However, some of the highly correlated bands remained prominently at the top of the violin plots referring to their elevated contributions, e.g., the B3 and B5 in the Sentinel-2 SSC model (Figure 7A) and B7 and B6 in the PlanetScope MPC model (Figure 7D). Remarkably, the contribution order of bands for the SSC model differed from its counterpart in the MPC model either in Sentinel-2 or PlanetScope satellites. Additionally, it also differed even for the same variable (i.e., SSC or MPC) comparing both satellites.

#### 3.3.3. Suspended Sediment-Based Models for Microplastic Concentration

Given the limited accuracy of sensors in directly estimating MPC, regression models between MPC and SSC were developed, leveraging their high correlation coefficients (Figure 5 and Table 3). The rough model which was built based on all spatiotemporal data demonstrated a moderate estimation accuracy (Table 3). Meanwhile, the estimation accuracy for the hydrologically individual models varied, ranging from low during low stages and falling limbs to very high during rising and peak phases (Table 3). The best fit of the models was achieved by the second- and third-degree polynomial regression lines in the peak and falling phases, while simple linear regression was the best in the rising phase.

### 3.4. Spatiotemporal Generalization Capability of the Developed Models

**Figure 8:**
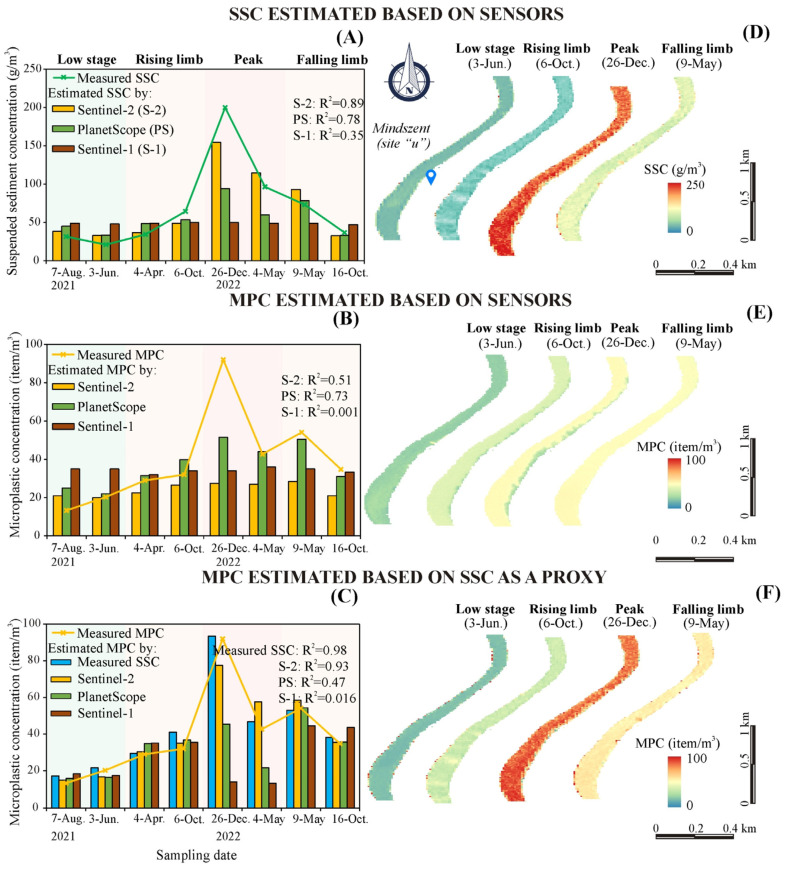
Performance of the suspended sediment concentration (SSC) and microplastic concentration (MPC) models through different hydrological conditions (i.e., low stage and rising, peak, and falling phases of floods) at Mindszent (site: ‘u’) (**A**–**C**), as well as their spatial distribution (**D**–**F**).

**Figure 9:**
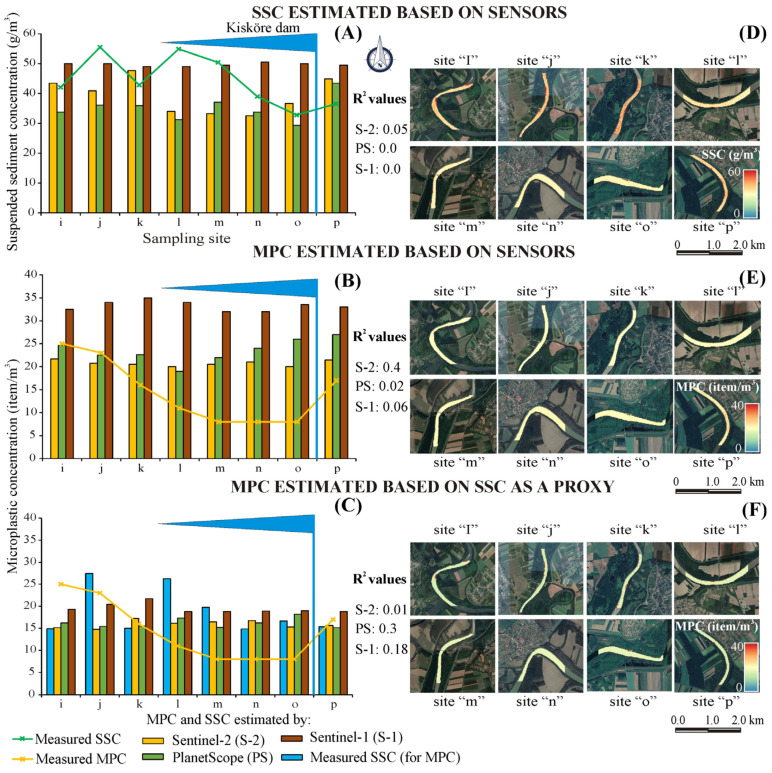
Performance of the suspended sediment concentration (SSC) and microplastic concentration (MPC) models along sites ‘i–p’ in the Middle Tisza (**A**–**C**), and their spatial distribution (**D**–**F**).

The spatiotemporal generalization capability of the developed SSC and MPC models was assessed (Figure 8 and Figure 9). The temporal generalization capability was evaluated by comparing their estimates with ground in situ measurements at Mindszent (site ‘u’), covering all hydrological scenarios (Figure 8A–C). In addition, the models with the best performance were utilized to reveal the longitudinal distribution of both variables during these periods (Figure 8D–F). The Sentinel-2 model gave the best estimation accuracy for SSC (R^2^ = 0.89) with very high sensitivity to the concentration changes during the various hydrological conditions (Figure 8A,D), meanwhile, the worst accuracy was achieved by the Sentinel-1 model (R^2^ = 0.35). The PlanetScope model demonstrated good estimation accuracy too (R^2^ = 0.78); however, it tends to underestimate the actual SSCs, particularly during peak periods.

Both approaches (models) of estimating MPC directly based on satellite sensors (Figure 8B) and indirectly through SSC (measured and estimated by satellites) as a proxy (Figure 8C) were tested. Concerning the direct method, the best estimates were achieved by the PlanetScope model (R^2^ = 0.73); however, it still underestimates the actual MPC, especially during peak periods (Figure 8B). Both the Sentinel-1 and Sentinel-2 MPC models showed low sensitivity to MPC changes, though the accuracy of the Sentinel-2 model (R^2^ = 0.51) was higher than the Sentinel-1 (R^2^ = 0.0). In the meantime, the indirect method proved its superiority over the direct method, as the R^2^ increased by 44.8% on average (Figure 8B,C). The estimated MPC based on the measured SSC gave the highest R^2^ (0.98). The best MPC estimates based on satellite-based SSCs were achieved by Sentinel-2 (R^2^ = 0.93) followed by PlanetScope (R^2^ = 0.47), while the lowest was by Sentinel-1 (R^2^ = 0.01) (Figure 8C).

The longitudinal distribution of the SSC and MPC seemed to be uniform along the monitoring site ‘u’ during the various hydrological conditions (Figure 8D–F). However, at some periods (e.g., the peak of flood wave; Figure 8E) precise MPC estimation on the eastern bank is impeded due to the shadow cast by trees in the floodplain.

The spatial generalization capability of the developed SSC and MPC models was evaluated by comparing their estimates with in situ measurements between sites “i” and “p” (July 2022) (Figure 9A–C). The best-performing models were employed to exhibit their spatial distributions at the designated sites (Figure 9D–F).

Most of the developed models performed badly with the spatial data (Figure 9). However, a favorable aspect is that the mean estimates of these models were in proximity to that of the ground in situ measurements, especially, in the case of the passive sensors. For instance, the mean measured SSC at the corresponding eight sites was 44.2 g/m^3^, and the mean estimates were 39.2 g/m^3^ (Sentinel-2), 35.1 g/m^3^ (PlanetScope), and 50.1 g/m^3^ (Sentinel-1) (Figure 9A). The presence of Kisköre Dam stimulated sediment deposition upstream (decline of SSC by 33.4% between sites ‘l’ and ‘o’) and clear water erosion downstream (increase by 21% at site ‘p’). This characteristic pattern was captured partially by the Sentinel-2 and PlanetScope sensors (Figure 9A,D), while it was totally missed by the Sentinel-1.

The two methods used to estimate MPC directly based on satellite sensors and indirectly through SSC as a proxy demonstrated similar but low estimation accuracies (Figure 9B,C). However, the indirect method gave mean MPC estimates (S-2: 15.9 item/m^3^; PS: 16.6 item/m^3^; S-1:19.5 item/m^3^) that were closer to the in situ measurements (14.5 item/m^3^) than the direct method (S2: 20.7 item/m^3^; PS: 23.5 item/m^3^; S1:33.25 item/m^3^). The measured MPs showed a similar characteristic pattern as of SSC in response to the Kisköre Dam since the concentrations declined by 27.3% between sites ‘i’ and ‘o’ and increased by 112.5% at site ‘p’. Similarly, this pattern was partially captured by the passive sensors and entirely missed by the active sensor.

The longitudinal distribution of SSC and MPC seemed to be uniform along the eight sites (i.e., ‘i’–‘o’) with some false alarms arising at the riverbanks, especially at point bars due to the shallow water at the measuring period (Figure 9D–F).

## 4. Discussion

### 4.1. Suspended Sediment (SS) and Microplastic (MP) Concentration Patterns in the Tisza River

The temporal variations in SSC and MPC in Mindszent and Szeged were strongly associated with the hydrological changes. The mean SSC in Szeged (66.8 g/m^3^) was 28% higher than in Mindszent (52 g/m^3^), due to the proximity of the Maros River to the sampling sites (Figure 1), as the Maros conveys large amount of suspended sediment load into the Tisza (8.3 million tons/year; [25]). Considering low stages, the SSC and MPC were negligibly correlated, probably because their sources are different: during dry periods most of the river water originates from groundwater inflow, whereas the MPs are probably emitted from WWTPs [4]. A similar negligible correlation during low stages was reported from the Langat River (Malaysia) [15]. Meanwhile, during flood waves, new sources that are common to both variables (e.g., surface runoff and remobilization of accumulated materials; [21,38]) appear in the system, leading to a very strong correlation. Focusing on flood phases, the lowest SSC and MPC, along with the weakest correlation were observed during the falling limb, potentially due to the attenuation of stream power and the accelerated deposition of sediment and MP as the river system returns to its pre-flood condition when the MP and SS originate from different sources [4].

Given the complex interaction of the river’s longitudinal variables (e.g., discharge, slope, flow velocity, locations of hydraulic structures and tributaries) the SSC and MPC revealed unclear downstream trends over time (i.e., August 2021 and July 2022). Moreover, the spatial distribution pattern of both variables differed at the section scale (i.e., S1–S5), indicating potential differences in their sources and/or transport mechanisms. However, it is noteworthy that these longitudinal samplings were conducted during dry periods when their correlation was negligible (Figure 5C). Notably, the distribution pattern of MPC showed a strong association with the waste management status of the sub-catchments, hence the increased MP contamination in the upper and lower reaches is likely attributed to insufficient wastewater management practices in Ukraine, NE Hungary, and Serbia [4]. On the other hand, the elevated SSC in the middle reach is likely associated with the main tributaries. A similar finding was reported in the Cinca River (Spain), as a four-fold increase in SSC was observed in the reach with tributaries [39].

Fibers dominated both the spatial (2021: 84.6%; 2022: 98%) and temporal (81%) measurements (Figure 2A,C,D), highlighting that wastewater is the main origin of MP contamination in the Tisza [21]. This finding was also reported in many other rivers worldwide, such as the Mulde and Elbe Rivers (Germany) [38] and the Langat River (Malaysia) [15]. Based on the level of MP pollution of the Tisza (temporal mean at Mindszent: 19.8 ± 14.2 item/m^3^), the river can be classified as moderately polluted compared to European rivers [3]. Meanwhile, globally, it is slightly polluted, as its concentration was significantly lower than the global median concentration (1.8 × 10^5^ item/m^3^) [40].

### 4.2. Efficiency of Satellite Sensors for Direct Estimation of Suspended Sediment and Microplastic Concentrations

#### 4.2.1. Evaluation of Passive Sensors

Passive sensors demonstrated good accuracy in estimating SSC (Figure 6A,B), consistent with previous studies [2,7,16]. However, the estimation accuracy was influenced by the characteristics of the employed sensor. This capability returns to the potential of SS particles to influence the reflectance received by a sensor, given its higher density and more uniform distribution in water compared to MPs. Meanwhile, these sensors showed remarkably low estimation accuracy for MPC (Figure 6D,E). This finding supports the hypothesis of Hu [9], who stated that the required concentration to be detected by satellite sensors is ≥600 items/m^2^, but the MPC in the Tisza is at least three times lower than this threshold. Furthermore, most MP particles are typically transported below the water surface or covered by a thin layer of water due to the turbulent dynamics of the river, making its detection by the visible and near-infrared bands significantly more challenging [9].

Despite the low MPC of the Tisza (2–129 item/m^3^), which prohibits its direct detection by satellite sensors, it is promising that the MPC showed a negligible to strong correlation with the various bands of Sentinel-2 and PlanetScope images (Figure 3A). This behavior indicates that the MPC may indirectly correlate with these bands through active water constitution (e.g., SSC, chlorophyll-a), as the correlation does not necessarily imply causation. Although this indirect correlation could offer some utility in estimating MPC using satellite sensors without a proxy, the developed MPC models in our study showed very low estimation accuracy (Figure 6D,E). Hence, we believe that utilizing Sentinel-2 or PlanetScope for estimating MPC directly may not be the most optimal choice, however, an indirect estimation using SSC data is promising.

Due to the trade-off between cost and accuracy, Sentinel-2 and PlanetScope offered two different combinations of satellite constellations exhibiting different accuracies for estimating SSC and MPC. The best-derived model for quantifying SSC was based on the Sentinel-2 sensor, potentially due to its higher SNR (Sentinel-2: 12–15 dB; PlanetScope: 10–12 dB) and wider bandwidth (mean of bands: Sentinel-2: 50 nm; PlanetScope: 31 nm) ([41]; Appendix A: Table A2), resulting in improved image quality. Moreover, the existence of additional red edge (B6 and B7) and NIR (B8) bands in Sentinel-2 may contribute to the enhanced estimation accuracy, since these bands demonstrated an elevated correlation with the SSC (Figure 3A).

Surprisingly, the best-performing model for MPC was achieved by PlanetScope; though, its estimation accuracy is still low (Figure 6E). This behavior could be explained by the higher spatial resolution of PlanetScope (3 m) and the consistency among its eight bands in contrast to the lower and different spatial resolutions of the Sentinel-2 bands. Although the Sentienl-2 bands were resampled, this adjustment could not substitute the higher level of details captured by the native high spatial resolution PlanetScope. The sub-pixel coverage of MP in PlanetScope was higher than Sentinel-2. However, this is still far from the required coverage to be detected by the satellite sensors [9], which explains the limited accuracy of the developed models. Also, the existence of the yellow band (B5) in PlanetScope, absent in Sentinel-2, may be an additional factor for the superiority of PlanetScope. This band has the highest reflectance difference between river and clear water (Figure 4B) and achieved the third-highest correlation coefficient with MPC (Figure 3A).

The SSC and MPC models demonstrated favorable generalization capability during the temporal testing (Figure 8), while it declined notably during the spatial testing (Figure 9). This discrepancy might be attributed to the fact that the temporal testing encompassed various hydrological conditions, while the spatial testing was limited to low stages. Therefore, additional longitudinal measurements during floods are necessary. During the temporal testing, the models yielded relatively accurate estimates of SSC and MPC during dry periods as well as the rising and falling phases. Meanwhile, it underestimated the concentrations during the peak phase (Figure 8A,B). This suggests that the models are slightly biased towards the lower concentrations due to the limited number of peak points used during the training process. To overcome this issue, denser temporal measurements during flood waves are required, as the peak phase is typically short. During the spatial testing, the models showed a mixture of a slight underestimation and overestimation of SSC and MPC (Figure 9A,B).

#### 4.2.2. Evaluation of Active Sensor

The Sentinel-1 exhibited disappointing findings, as the VV and VH channels had negligible correlation with the SSC and MPC, besides the developed models performed badly. This finding opposes our hypothesis that Sentinel-1 can estimate the SSC/MPC employing the correlation between the dielectric constant changes and suspended particulate material content. Probably, the SSC/MPC in the Tisza is not sufficiently high to influence the dielectric constant of water. This assumption is consistent with the findings of Shao et al. [32], who stated that the SSC and Sentinel-1 channels are linearly correlated, but only for values ≥1200 g/m^3^. In contrast, the maximum recorded SSC in the Tisza during the studied period was 322.5 g/m^3^. Therefore, further studies in rivers with various turbidity are required for a thorough evaluation of this approach. Another approach should be devised for the lowland rivers with medium SSCs.

The low observed correlations in the Tisza could also indicate that the surface roughness might have a higher impact on the backscattering of the VV and VH channels than the dielectric constant. This can be inferred from the variability of channels’ backscattering even for very similar SSC/MPC (Figure 3D), which is more likely to be attributed to the surface roughness changes. Particularly, the temporal monitoring site at Mindszent is situated in a port, and the measurements were performed on a ferry; hence, its movements stimulate water turbulence and affect the surface roughness condition. Accordingly, investigating the impact of SSC/MPC on surface roughness may prove valuable in estimating their concentrations using active sensors in rivers.

### 4.3. Applicability of Neural Network Algorithm for Developing Microplastic and Suspended Sediment Concentration Models

The MLP neural network algorithm has demonstrated its high degree of potential to estimate SSC/MPC in rivers (especially SSC) by the reflectance of optical sensors, consistent with the findings of Mohsen et al. [7] and Umar et al. [42]. The strength of this algorithm lies in its capability to learn the complex and non-linear relationships between features [33], which is adequate to model the non-linearly correlated SSC/MPC and reflectance of optical sensors (Figure 3B,C). The accuracy of the developed MLP neural network models for SSC (R^2^ = 0.71) in the Tisza was comparable to that in the Missouri and Mississippi Rivers (USA) (R^2^ = 0.72) [42], and the Yangtze River (China) (R^2^ = 0.72) [43]. However, it was higher than the models developed based on linear regression in the Maumee (R^2^ = 0.56) and the Mississippi Rivers (USA) (R^2^ = 0.62) [44].

Although applying the Optuna library to fine-tune the models’ hyperparameters was computationally expensive, it was very efficient for obtaining the best combinations. This is attributed to the implementation of Bayesian optimization techniques within the library, which could find the best hyperparameters without the need to exhaustively explore the entire search space [36]. Also, the SHAP library was very efficient for interpreting the models’ performance by providing insights into the contribution of each band through SHAP values. Notably, the order of the most contributed bands did not align precisely with the order of the most correlated bands to SSC/MPC (Figure 3A and Figure 7). This discrepancy might be linked to the MLP neural network nature that explores higher-order interactions between bands beyond the pairwise relationships captured by the correlation coefficient [33].

### 4.4. Evaluation of Indirect Estimation of Microplastic by a Proxy: Advantages and Limitations

The indirect estimation of MPC in rivers using active water constituents (e.g., SSC) as a proxy is likely the optimal solution. This can be inferred from the elevated estimation accuracy of this method (Table 3) compared to the direct method (Figure 6D–F) since the indirect method could circumvent the radiometric and spatial limitations of the satellite sensor which hampers direct detection of MPs [9]. This approach can be implemented relying on either in situ measurements or satellite estimates of the proxy. Although the former technique provides relatively high accuracy, it has limited applicability to the specific sampling site; meanwhile, the latter technique strikes a balance between accuracy and spatiotemporal coverage.

The SSC is a reliable proxy for MPC in rivers, especially during floods, consistent with the previous studies [2,15,16]. However, we report some limitations: (1) the SSC has low sensitivity to the MPC changes during low stages, though it can still provide reasonable estimates (but not highly accurate); (2) the established relationship is highly sensitive to sudden changes in the sources of sediment and/or MP either in space or time; hence their relationship needs to be calibrated recurrently; (3) the derived SSC-MPC relationship needs to be calibrated at confluences, dams and WWTP effluents.

### 4.5. Implications of the Developed Microplastic Models and Future Research

The developed MPC models based on SSC as a proxy, estimated by satellites, provide an efficient and cost-effective method to monitor MP transport in rivers. This would enhance our understanding of MP sources, pathways, and sinks, as well as assess the impact of alterations in land use and extreme events on its transportation. Furthermore, these models would serve as valuable tools to evaluate the efficiency of WWTPs and help water resource managers in evaluating their mitigation plans [45].

Though our findings provide valuable insights into the capabilities of different passive and active sensors to estimate MPC in rivers, additional research is needed. More research efforts should be geared towards proxy models owing to the resulting low estimation accuracy of the direct models. The models were validated longitudinally based on measurements collected only during low stages, hence, additional longitudinal measurements during floods are required to obtain a comprehensive evaluation. Further SSC and MPC measurements performed specifically in the vicinity of confluences, dams, and WWTP effluents are needed to validate their correlation at these spots. Additional research is needed to explore the pertinence of other water quality parameters (e.g., chlorophyll-a and colored dissolved organic matter) as potential proxies for MP. Integrating these influential parameters with SSC would enhance the estimation accuracy and overcome the limitations of SSC during the low stages.

## 5. Conclusions

The remote sensing of MPC in aquatic environments, especially in rivers, is still in its nascent stage of research, with conflicting findings regarding its applicability. However, this approach holds great potential for enhancing our understanding of MP transport in rivers and assisting decision-makers in devising effective mitigation scenarios. Our study investigated the potential of various passive (i.e., Sentinel-2 and PlanetScope) and active (Sentinel-1) satellite sensors to estimate MPC in the medium-sized, lowland Tisza River, based on intensive SSC and MPC spatiotemporal measurements. Two approaches were evaluated, including the direct estimation of MPC through the reflectance/backscattering of the satellite sensors, and indirect estimation using SSC as a proxy. In both cases, MLP neural network-based models were developed for the SSC/MPC.

The Tisza is slightly (globally) to moderately (among European rivers) contaminated by MPs, and the treated or untreated wastewater is the main source of MPs. The direct estimation of MPC by satellites presents significant challenges due to limitations in their radiometric and spatial capabilities coupled with low MP abundance in rivers (in our case ≤129 item/m^3^). Alternatively, the indirect estimation by SSC as a proxy is a promising approach, as its estimation accuracy (R^2^) for MPC could reach 0.88 (during the peak phase). However, it is noteworthy that the complex relationship between MP and SS transport introduces some limitations. For instance, during low stages, SSC may provide inaccurate estimates of MPC; moreover, their established relationship at a particular site should be validated longitudinally, especially at the locations of confluences, dams, and effluents of WWTPs.

The resolutions and technical characteristics of the employed sensors affected its estimation accuracy for SSC/MPC. Accordingly, the Sentinel-2 with its higher spectral bands, SNR, and wider bandwidth outperformed PlanetScope for estimating SSC and subsequently MPC. However, PlanetScope with its daily revisit frequency provided more frequent SSC/MPC data, though with lower accuracy. On the other hand, Sentinel-1 was inadequate for estimating SSC/MPC directly based on backscattering signals.

Although the MPC was estimated by two approaches in this study, the estimation accuracy still requires further enhancements for better predictions during different hydrological scenarios. Additional research considering the surface roughness of river water as a proxy for MPC (in the case of active sensors) is warranted, given the potential of these sensors to capture roughness accurately. Additionally, integrating other water quality parameters into the SSC-MPC relationship requires further investigation. This would enhance the estimation accuracy and overcome the limitations of SSC as a proxy. These efforts will contribute to improving the precision and pertinency of remote sensing techniques for monitoring SSC/MPC in rivers.

## Figures and Tables

**Table 2:** Fine-tuned hyperparameters leveraged by Optuna for training/validating suspended sediment (SS) and microplastic (MP) concentration models based on Sentinel-2, PlanetScope, and Sentinel-1 sensors.

Model		Hyperparameters						
		Hidden Layer Sizes	Activation Function	Solver	Alpha	Learning Rate	Maximum Iteration	Batch Size
SSC	Sentinel-2	(100, 5, 20)	logistic	lbfgs	0.0003	invscaling	604	67
	PlanetScope	(40, 10, 20)	relu	lbfgs	0.0239	constant	669	120
	Sentinel-1	(50, 50)	tanh	adam	0.0001	constant	521	44
MPC	Sentinel-2	(100)	relu	adam	0.0191	invscaling	985	33
	PlanetScope	(150, 150)	tanh	adam	0.0001	adaptive	865	121
	Sentinel-1	(10, 15)	relu	sgd	0.0011	adaptive	748	116

**Table 3:** Microplastic (MP) concentration models based on suspended sediment concentration (SSC) as a proxy considering all hydrological conditions and individually (i.e., low stage and rising, peak, and falling phases of floods).

Hydrological Condition	Regression Model	R^2^	RMSE (Item/m^3^)	MAE (Item/m^3^)
All hydrological conditions	MPC =−0.0004 SSC^2^ + 0.499 SSC + 8.790	0.51	17.9	13.7
Low stage	MPC = 0.001 SSC^3^ − 0.093 SSC^2^ + 2.193 SSC + 6.902	0.17	12.9	9.4
Rising limb	MPC = 0.391 SSC + 15.942	0.83	11.8	9.5
Peak	MPC = −0.0017 SSC^2^ + 0.954 SSC-29.445	0.88	7.76	10.76
Falling limb	MPC = 6 × 10^−5^ SSC^3^ − 0.014 SSC^2^ + 1.425 SSC + 2.278	0.28	22.8	18.18

## Data Availability

The data and the implemented code in this study can be accessed at the following repository: https://github.com/AMohsenMetwaly/Remote-sensing-of-microplastic (accessed on 2 October 2023).

## Appendix A

**Table A1:** Typical hydraulic parameters, sediment, and microplastic (MP) concentration characteristics of the various sections of the Tisza River (S1–S5) (data source: [4,19,21,46]).

Tisza Reach	Upper		Middle		Lower
Section	S1	S2	S3	S4	S5
Slope (cm/km)	2000–5000	110–13	≥ 3	1–3	<2.5
Flow velocity (m/s)	2–3	1	0.1–0.5	0.1–0.2	<0.2
Mean discharge (m^3^/s)	22	207	346	509	864
Bed load (m^3^/year)	No data	22.6 × 10^3^	8.8 × 10^3^	11 × 10^3^	9 × 10^3^
Suspended load (m^3^/year)	No data	0.9 × 10^6^	5 × 10^6^	12.2 × 10^6^	12.9 × 10^6^
Mean SSC (g/m^3^)	No data	74	85	97	384
Mean MPC in the water (item/m^3^) (during a low stage)	39 ± 31.1	18.6 ± 14.2	15.8 ± 13.8	14.5 ± 7.9	22.6 ± 10.1
Mean MPC in the sediment (item/dry kg) (during a low stage)	978 ± 817	1625 ± 1223	2082 ± 1511	2019 ± 1305	530 ± 169

**Table A2:** The spectral and spatial characteristics of the Sentinel-2 and PlanetScope bands.

Spectral Band	Sentinel-2			PlanetScope		
	Central Wavelength (nm)	Spatial Resolution (m)	Bandwidth (nm)	Central Wavelength (nm)	Spatial Resolution (m)	Bandwidth (nm)
B1	443 ^a^	60	20	443 ^a^	3	21
B2	492 ^b^	10	65	490 ^b^	3	50
B3	560 ^c^	10	35	531	3	36
B4	665 ^d^	10	30	565 ^c^	3	36
B5	704 ^e^	20	15	610	3	20
B6	741	20	15	665 ^d^	3	30
B7	783	20	20	705 ^e^	3	16
B8	833	10	115	865 ^f^	3	40
B8a	865^f^	20	20	–	–	
B9	945	60	20	–	–	
B10	1374	60	30	–	–	
B11	1614	20	90	–	–	
B12	2202	20	180	–	–	

Alphabetical letters indicate the compatible bands of both satellites.
